# A lab-on-a-chip for rapid miRNA extraction

**DOI:** 10.1371/journal.pone.0226571

**Published:** 2019-12-19

**Authors:** Ole Behrmann, Matthias Hügle, Peter Bronsert, Bettina Herde, Julian Heni, Marina Schramm, Frank T. Hufert, Gerald A. Urban, Gregory Dame

**Affiliations:** 1 Department of Microbiology and Virology, Brandenburg Medical School Fontane, Neuruppin, Germany; 2 Laboratory for Sensors, Department of Microsystems Engineering (IMTEK), University of Freiburg, Freiburg, Germany; 3 Institute for Surgical Pathology, Medical Center–University of Freiburg, Freiburg, Germany; 4 Faculty of Medicine, University of Freiburg, Freiburg, Germany; 5 German Cancer Consortium (DKTK), Partner Site Freiburg, Freiburg, Germany; University of Helsinki, FINLAND

## Abstract

We present a simple to operate microfluidic chip system that allows for the extraction of miRNAs from cells with minimal hands-on time. The chip integrates thermoelectric lysis (TEL) of cells with native gel-electrophoretic elution (GEE) of released nucleic acids and uses non-toxic reagents while requiring a sample volume of only 5 μl. These properties as well as the fast process duration of 180 seconds make the system an ideal candidate to be part of fully integrated point-of-care applications for e.g. the diagnosis of cancerous tissue. GEE was characterized in comparison to state-of-the-art silica column (SC) based RNA recovery using the mirVana kit (Ambion) as a reference. A synthetic miRNA (miR16) as well as a synthetic snoRNA (SNORD48) were subjected to both GEE and SC. Subsequent detection by stem-loop RT-qPCR demonstrated a higher yield for miRNA recovery by GEE. SnoRNA recovery performance was found to be equal for GEE and SC, indicating yield dependence on RNA length. Coupled operation of the chip (TEL + GEE) was characterized using serial dilutions of 5 to 500 MCF7 cancer cells in suspension. Samples were split and cells were subjected to either on-chip extraction or SC. Eluted miRNAs were then detected by stem-loop RT-qPCR without any further pre-processing. The extraction yield from cells was found to be up to ~200-fold higher for the chip system under non-denaturing conditions. The ratio of eluted miRNAs is shown to be dependent on the degree of complexation with miRNA associated proteins by comparing miRNAs purified by GEE from heat-shock and proteinase-K based lysis.

## Introduction

MicroRNAs (miRNAs) are a family of more than 600 short single-stranded RNA molecules [1]. MiRNAs alter gene expression by hybridization to specific mRNA sites and are associated with a multitude of cellular functions [2].

Dysregulation of miRNAs has been shown to play a pivotal role in the pathogenesis of a multitude of cancers [3] and chemoresistance [4]. Thus, measurement of the relative amounts of miRNAs extracted from cells can be used as a biomarker for diagnosis and classification of neoplasms [5].

Cellular miRNA patterns are typically derived in a three-step process. First, cells are lysed by mechanical and/or chemical action. Released miRNAs are then purified from cellular debris and other co-released nucleic acids by adsorption to a silica surface, most commonly in the form of either spin-columns or silica-coated magnetic beads[6,7]. After release from the surface, specific miRNAs are detected by sequencing [8] or amplification techniques such as stem-loop RT-qPCR [9–12].

However, most of these macroscopic purification systems require sample volumes in the milliliter range as well as a multitude of manual handling and pipetting steps[13]. Additionally, a potentially harmful mixture of phenol and chloroform is used for phase separation of DNA, RNA and proteins prior to the silica surface adsorption step[14].

A specific challenge in the analysis of miRNA patterns is the fast isolation of miRNAs from low amounts of cells [15]. Even though this problem lends itself to a possible solution using microfluidic technology, few approaches have been published. Schoch et al. [16] demonstrated the isolation of small RNA species from 293A human kidney cell lysates by on-chip isotachophoresis (ITP) from a sample volume of 5 μl. The detection limit was found to be ~900 cells. Shintaku et al. [17] present the extraction of total RNA and DNA from single cells inside a microfluidic channel. Total RNA was detected by non-selective intercalation with SYBR Green II dye.

**Fig 1:**
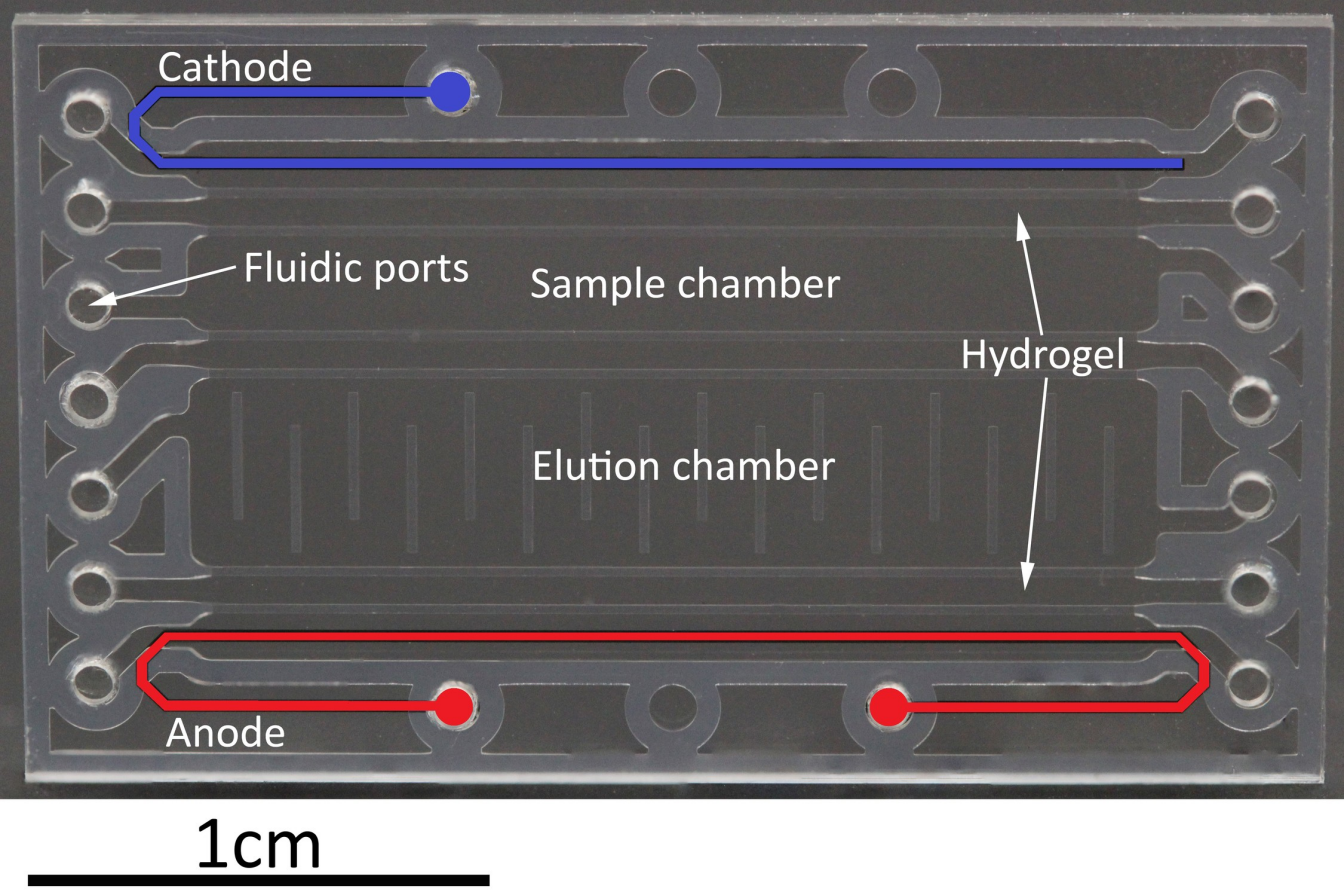
Chip system used for miRNA extraction. The sample chamber (5 μl) and elution chamber (10 μl) are separated by a hydrogel. The cathode (blue) and anode (red) are inside fluidic channels that are electrically connected to the sample and elution chambers by hydrogels. Controlled filling and emptying of the elution chamber is facilitated by capillary pressure barriers.

We present a novel microfluidic approach that combines cell lysis and elution of released miRNAs in a single device. From the principles first put forth by the work of Vulto et al.[18] and Dame et al.[19], we have developed a simple microfluidic chip (see Fig 1) that allows for the extraction of miRNAs from only five cancer cells within three minutes while requiring a sample volume of just 5 μl. The chip is based on unique microfluidic technology that enables controlled and bubble free loading of high aspect-ratio microfluidic chambers by means of hydrodynamic pressure barriers (“phaseguides”)[20].

**Fig 2:**
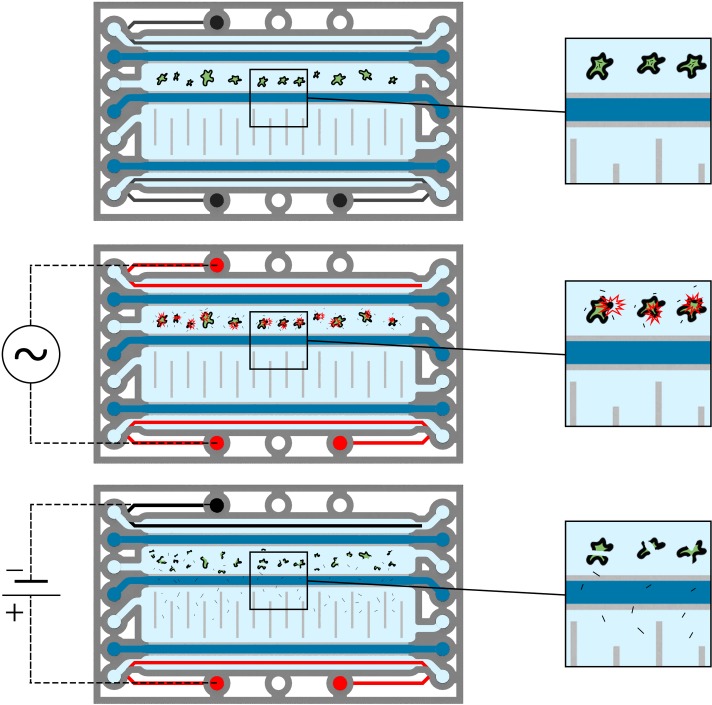
**Illustration of the process for microfluidic miRNA extraction (top view). Top:** Cells are introduced into the sample chamber. **Center:** A high frequency voltage is applied between the two coplanar electrodes (red)–cell lysis is caused by joule heating of the liquid sample. **Bottom:** A DC voltage is applied to the coplanar electrodes. Anionic miRNAs migrate through the separation hydrogel (dark blue) towards the anode (red) and are collected in the elution chamber. Large nucleic acids and non-polar cell debris remain inside the sample chamber.

Here, phaseguides are used to construct microfluidic chambers that are interconnected by hydrogels (see Figs 1 and 2). Cells are introduced into the sample chamber (sc) and are lysed by a high frequency electric field applied by coplanar electrodes. Subsequently, released miRNAs are eluted from the crude lysate by on-chip gel-electrophoresis into the elution chamber (ec).

In this work, MCF7 breast cancer cells are used as a biological model to test and characterize the novel microfluidic chip system. As both tumor associated miRNA biomarkers as well as endogenous control RNAs need to be quantified for clinical diagnosis, target sequences from both classes are quantified by stem-loop RT-qPCR. For the tumor associated RNA, we chose hsa-let-7a-5p (Abbr.: let7a, [21]) and as an endogenous control hsa-mir-16-5p (Abbr.: miR16, [22]). As a further endogenous control, we also detect the small nucleolar RNA SNORD48.

## Results

### Characterization of miRNA elution behavior

**Fig 3:**
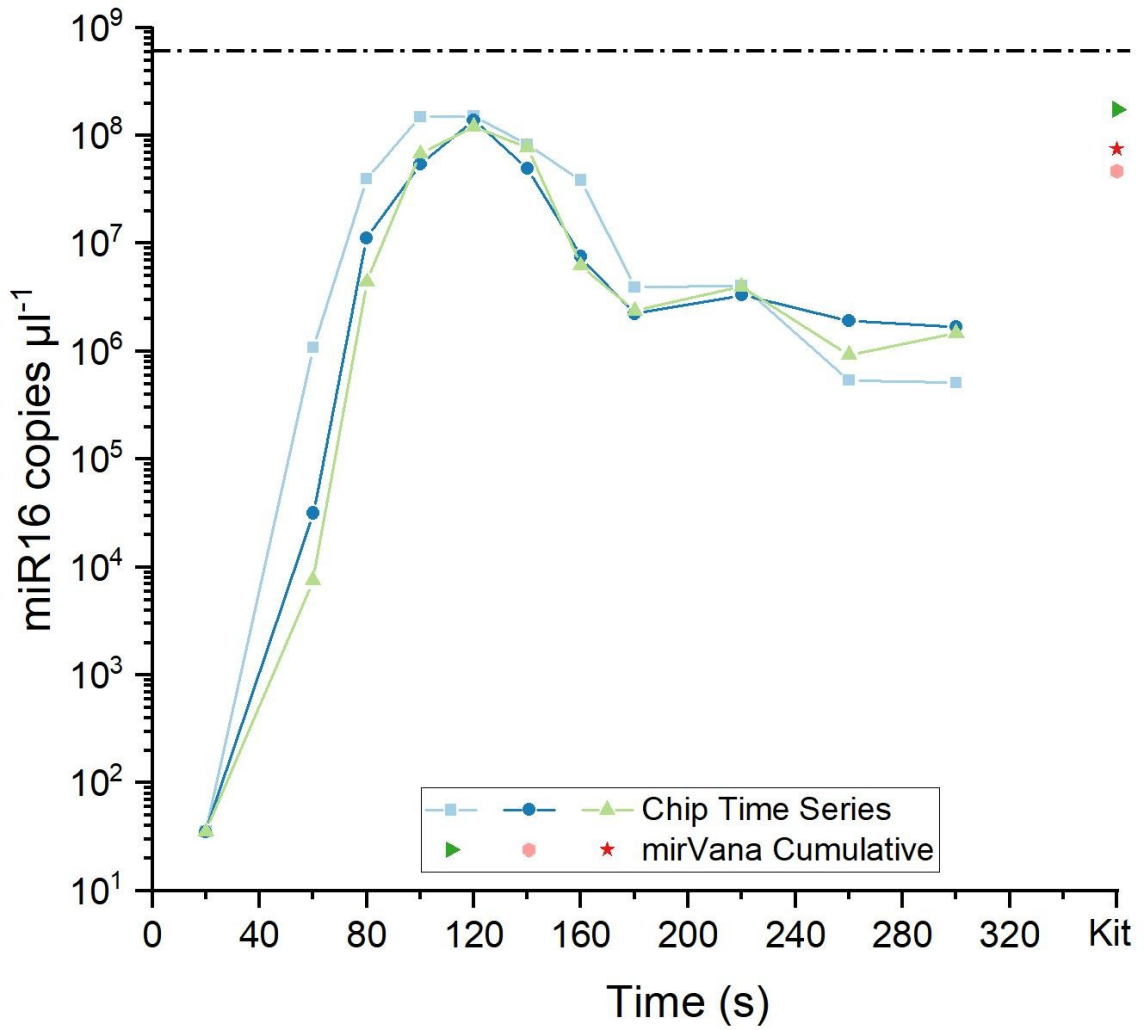
Comparison of GEE performance with mirVana miRNA extraction kit. Solid lines: GEE elution profile of miR16. Single points: miR16 extracted with the mirVana kit. Dashed line: Concentration of the input sample for GEE and kit extraction.

As presented in Fig 3, the majority of the initial synthetic miR16 sample (6.02*10^8^ μl^-1^) was recovered from the elution chamber in a narrow window centered around the 140 second fraction. Elution continued at a strongly reduced rate until the end of the experiment at 300 seconds. The fractions taken at 100, 120 and 140 seconds each yielded miR16 concentrations comparable to those achieved by the mirVana kit extraction procedure, showing higher recovery performance of GEE in comparison to the kit. However, the exact miRNA concentration that is recovered from the chip is dependent on the time point at which the eluted fraction is recovered from the chip. For example, if a first sample is taken at 80 seconds, a subsequent sample taken at 140 seconds will contain a higher miRNA concentration than a sample taken at 100 seconds.

**Fig 4:**
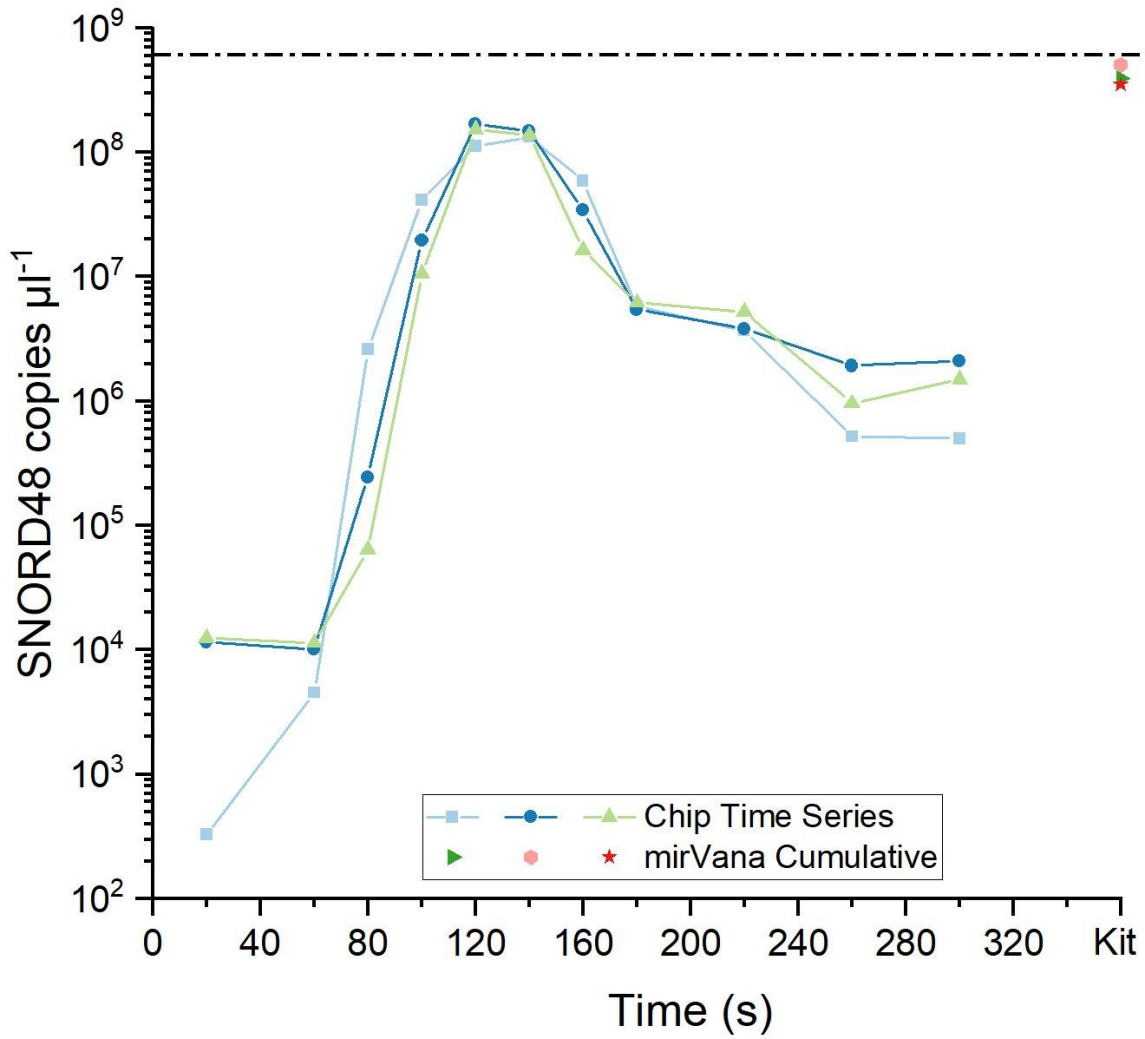
Comparison of GEE performance with mirVana miRNA extraction kit. Solid lines: GEE elution profile of SNORD48. Single points: SNORD48 extracted with the mirVana kit. Dashed line: Concentration of the input sample for GEE and kit extraction.

The overall elution profile for SNORD48 (Fig 4) is similar to that of miR16. However, the elution peak is broadened with almost equal amounts being eluted into the 140 and 160 second fractions. This is expected as the greater length of SNORD48 means that it will migrate slower through the hydrogel. The two main fractions each contained lower amounts of SNORD48 than was recovered by the mirVana kit procedure. Again, the eluted amount of RNA is highly dependent on the time point at which the fraction is taken. Another possible reason for the better performance of the kit for SNORD48 in comparison to miR16 is that the longer RNA may have better binding/elution characteristics on silica columns.

### Cell lysis

Lysis duration is limited by formation of gas-bubbles inside the microfluidic chambers. These bubbles form due to nucleate boiling of the suspension medium. After 30 seconds bubble formation increased significantly leading to the loss of a significant part of the liquid inside the chip. Based on this observation, a lysis duration of 30 seconds was chosen for further experiments.

**Fig 5:**
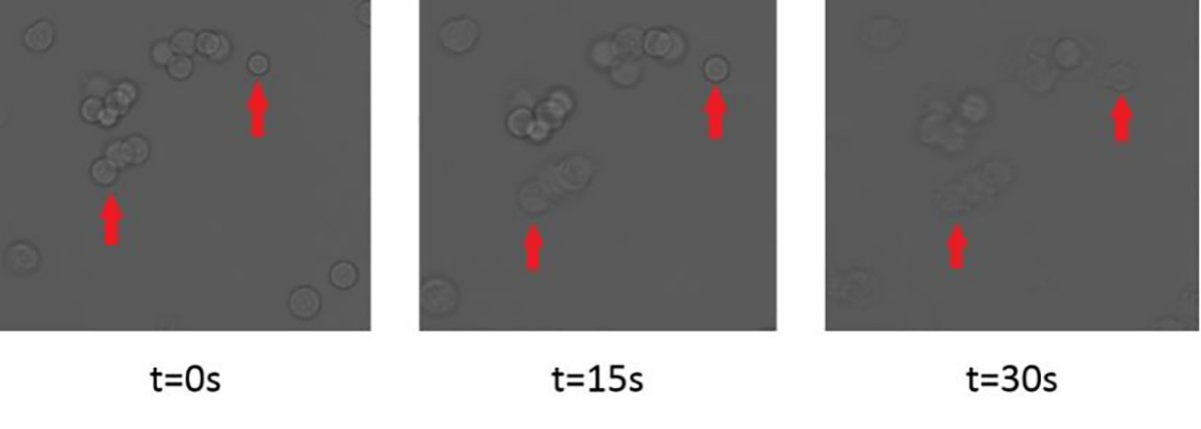
Micrograph of the thermo-electric lysis of MCF7 cells inside the chip. A 230 V_pp_ sine wave at a frequency of 20 kHz was applied to the coplanar chip electrodes for 30 sec. Disintegration of the cell membranes became evident after 15 sec. After 30 sec., the structural integrity of the cell membranes was disrupted.

Thermo-electric lysis of MCF7 cells was confirmed by microscopy (Fig 5). Cell membranes started to become indistinct and less well defined after 15 seconds (indicated by arrows in Fig 5). It is expected that release of cytoplasmic miRNAs already begins at this stage. After 30 seconds, most cells have disintegrated and have released parts of their cytoplasm into the suspension medium.

### Elution of miRNAs from crude lysate

**Fig 6:**
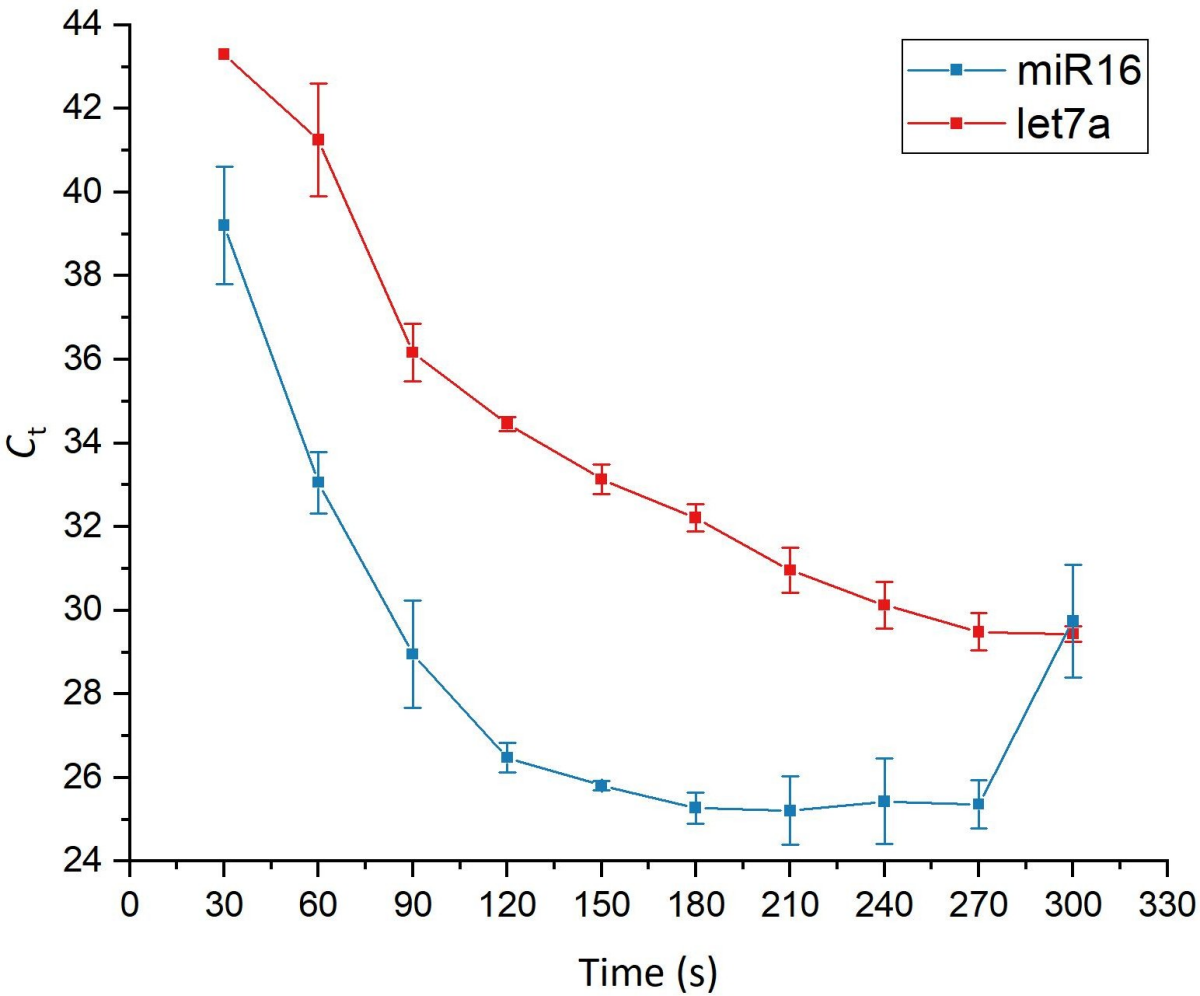
Time series for the extraction of miRNAs from 5000 MCF7 cells. Black: miR16; Red: let7a (n = 3).

Released miRNAs were eluted from the raw lysates by on-chip gel-electrophoresis. To determine the optimal actuation duration, a time series was conducted (see Fig 6). Electrophoresis was stopped every 30 sec. and the contents of the elution chamber were retrieved. The elution chamber was then refilled with fresh 1X TBE buffer and electrophoresis was continued for another 30 sec. up to a maximum of 300 sec. Both miR16 and let7a were detected by stem-loop RT-qPCR in the collected fractions.

**Fig 7:**
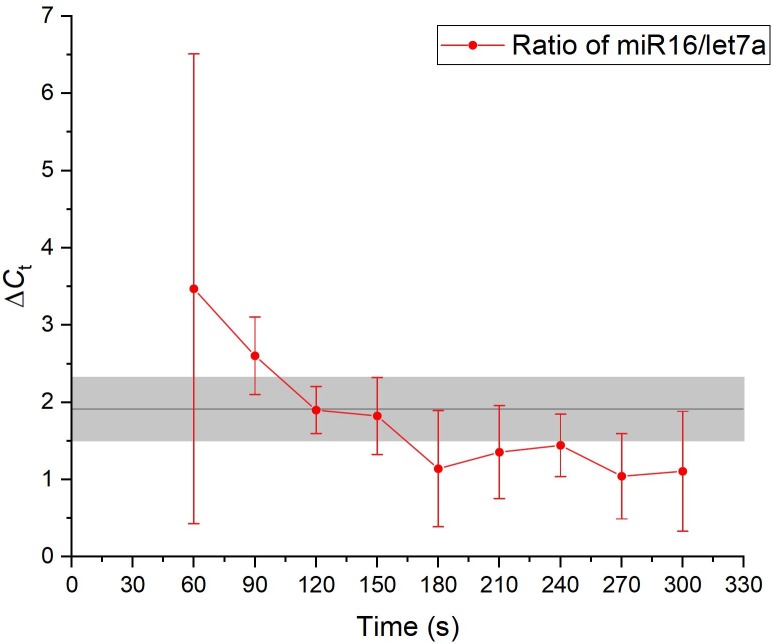
Time series for the on-chip gel-electrophoresis of kit extracted miRNA. The gray area denotes the ratio of miR16 to let7a in the input sample. The red data points are the ratio detected in the eluted fractions. Both the fractions at 120s and 150s display a ratio that is very close to the ratio of the input sample. This observation indicates that both miRNA species are eluted at the same rate (n = 3).

The amount of eluted miR16 increased steadily until constant elution was reached after about 150 seconds. Elution began to terminate after about 270 seconds, where a sudden sharp decrease in the eluted amount was observed. In comparison, the elution profile of let7a was shifted upwards due to the lower abundance of let7a (Fig 6). However, constant elution was not reached within the observed timeframe of 300 seconds and the overall shape of the elution curve was different in comparison to that of miR16. This result was quite unexpected, as both miRNAs are of the same length and would thus be expected to migrate at the same rate (if no secondary or higher structures exist) during gel-electrophoresis. To verify that the observed behavior was indeed due to the nature of the sample and not caused by the chip system, a kit purified miRNA sample was subjected to on-chip gel-electrophoresis. Again, a time series was performed with fractions being analyzed every 30 sec. The ratio of miR16 and let7a detected in the eluted fractions was then determined by stem-loop RT-qPCR and compared to the ratio in the initial sample (Fig 7). Fractions eluted after 120 seconds and 150 seconds, respectively demonstrated ratios very close to the ratio of the input sample. This observation indicates that the elution patterns obtained for the extraction of miRNAs from MCF7 cells are intrinsic to the sample and are not caused by the on-chip gel-electrophoresis.

**Fig 8:**
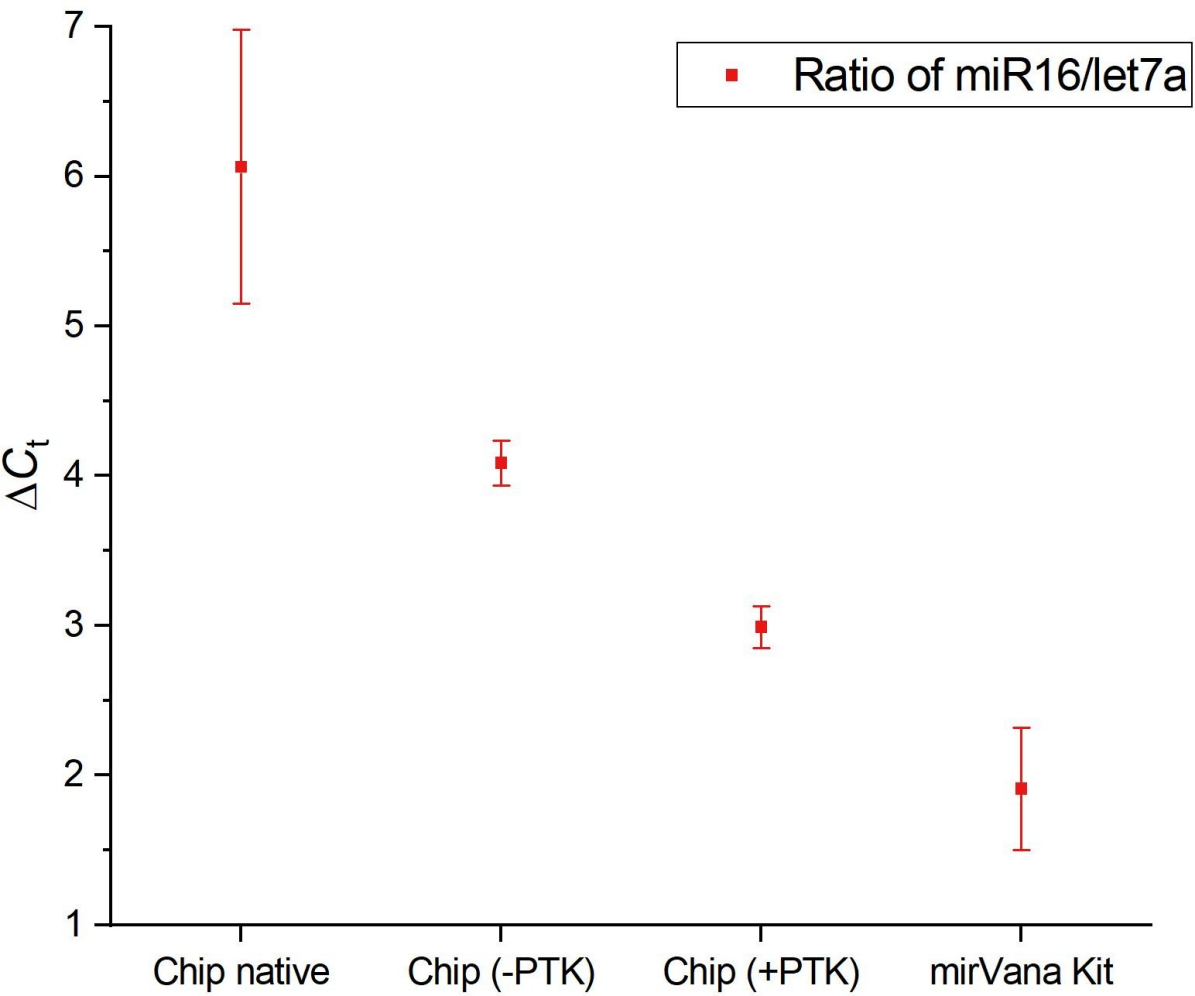
Comparison of miR16/let7a ratios. Chip native: Thermo-electric on-chip lysis and gel-electrophoresis. Chip (-PTK): Off-chip lysis by heat shock and on-chip gel-electrophoresis of the crude lysate. Chip (+PTK): Off-chip lysis by heat shock, proteinase K digestion and on-chip gel-electrophoresis of the digestion product. mirVana Kit: miRNA extracted by the mirVana kit (n = 3).

To test whether protein complexation of miRNAs is influencing GEE, MCF7 cells were lysed outside of the chip by heat shock and the resulting lysate was either directly subjected to on-chip gel-electrophoresis or first digested by proteinase K to degrade miRNA associated proteins. The resulting 150 second fractions were analyzed for their miR16/let7a ratios and compared to the ratios obtained by on-chip thermoelectric lysis and for kit extracted miRNA (Fig 8). TEL results in the highest miRNA rations, whereas miRNA obtained by the mirVana kit procedure results in the lowest ratios. While lower than the ratio obtained for lysis by thermal shock, the miRNA ratio obtained for lysis by thermal shock followed by proteinase K treatment is higher than that for kit extracted miRNA. These results show that TEL is the most gentle of the investigated lysis methods and releases miRNAs in a more native state while still complexed with miRNA-associated proteins.

### Comparison to commercial kit

To compare the performance of the chip system to that of a commercial extraction kit (mirVana, Ambion), an electrophoresis duration of 150 seconds was used.

**Fig 9:**
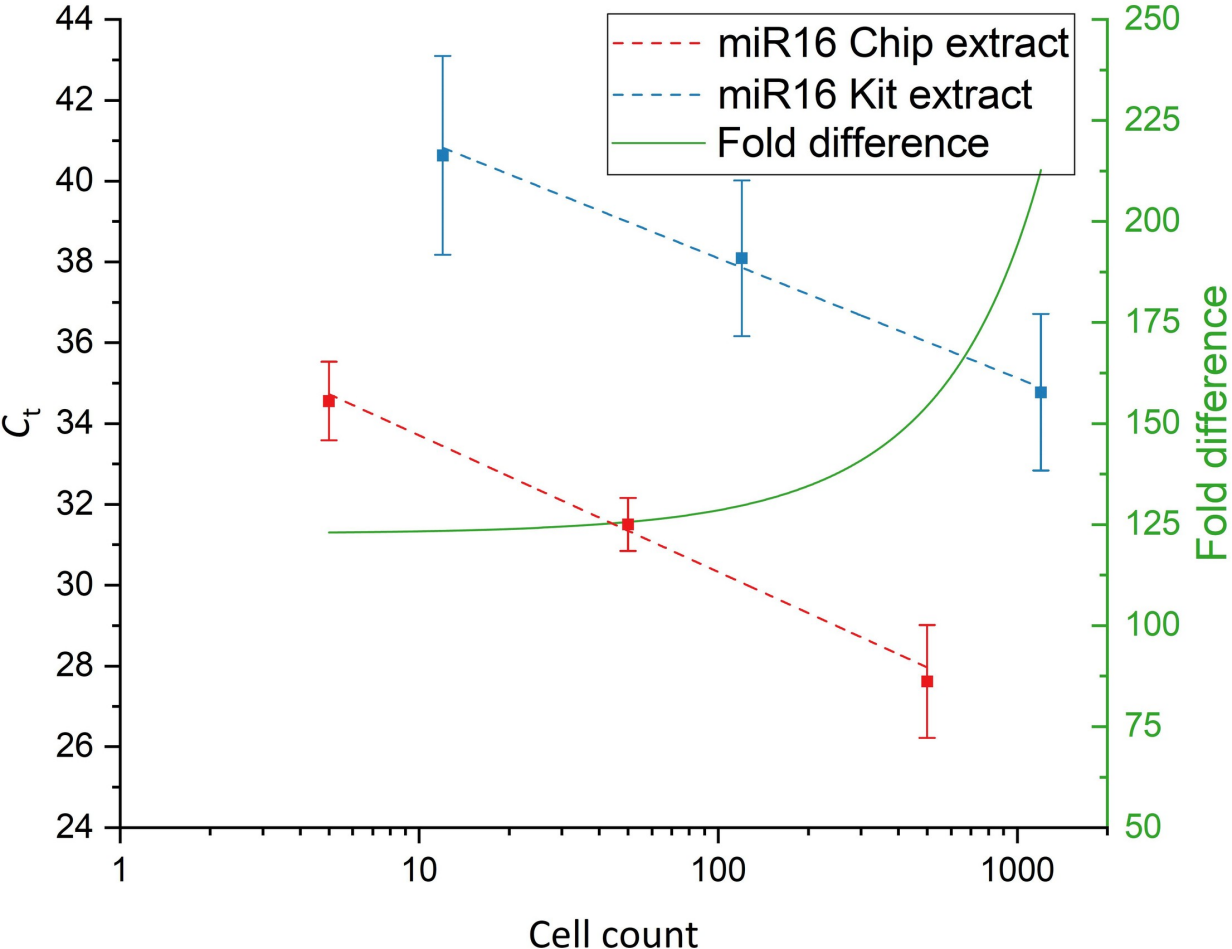
Analysis of MCF7 extracts for miR16. Red: Chip extracts; Blue: Kit extracts. Linear fits (dashed lines) are applied to the C_t_ plots and the difference in detected miRNA concentrations was calculated by taking the differences of the linear fits (green, right axis) (n = 3).

**Fig 10:**
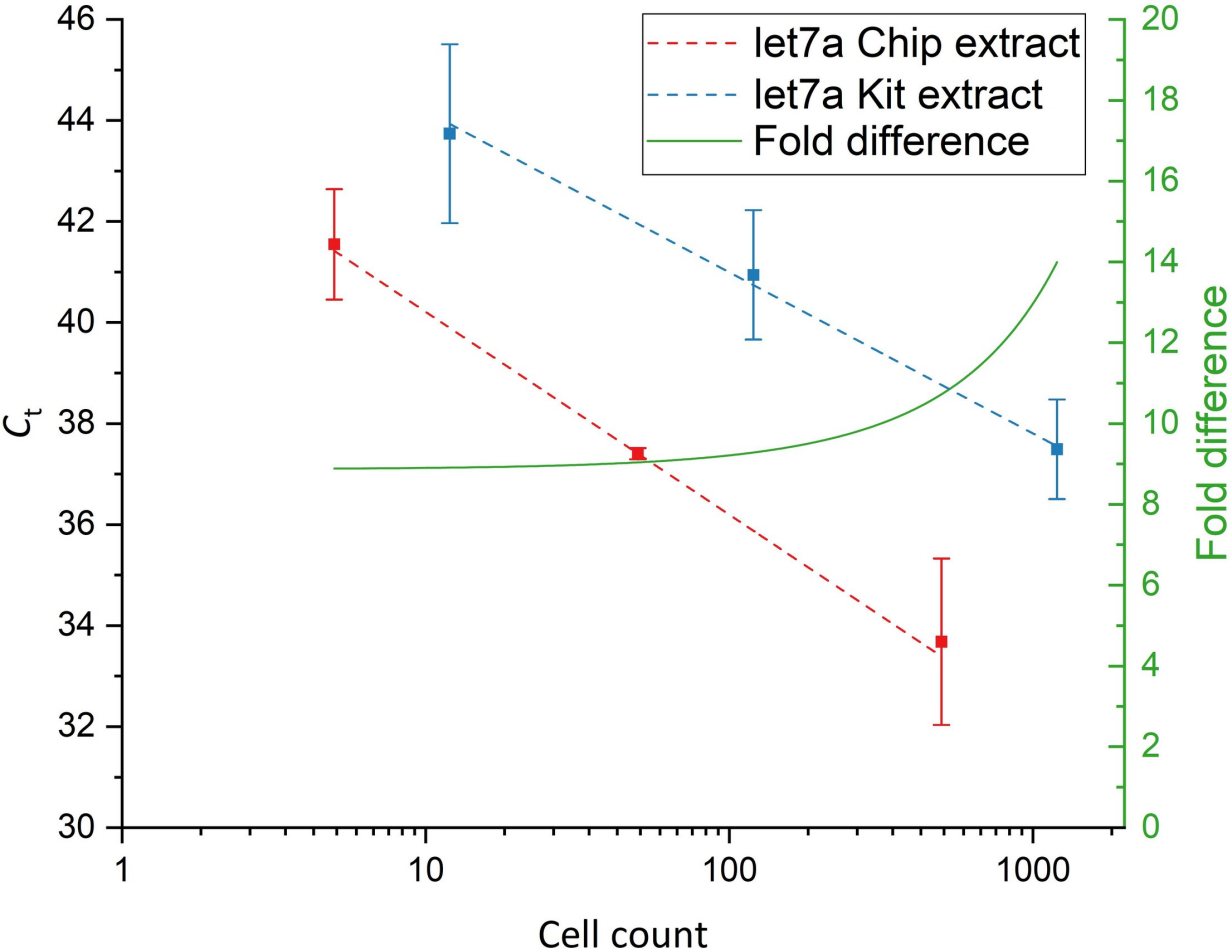
Analysis of MCF7 extracts for let7a. Red: Chip extracts; Blue: Kit extracts. Linear fits (dashed lines) are applied to the C_t_ plots and the difference in detected miRNA concentrations was calculated by taking the differences of the linear fits (green, right axis) (n = 3).

Figs 9 and 10 shows the performance of the chip system in comparison to the commercial kit for the extraction of miRNAs from serial dilutions of MCF7 cells. Both miR16 and let7a were successfully detected by on-chip extraction in samples containing as few as 5 cells total. Both the chip and the commercial kit displayed linear extraction behavior over the complete range of tested dilutions.

As expected, the detected levels differed for both investigated miRNAs, with miR16 showing a higher abundance than let7a. However, the extraction performance of the chip system relative to the kit was found to be dependent on the investigated miRNA.

For miR16, the chip performs about 110- to 220-fold better than the kit whereas for let7a the chip performed between 8.5- and 14-fold better.

### Extraction of a small nucleolar RNA (snoRNA)

**Fig 11:**
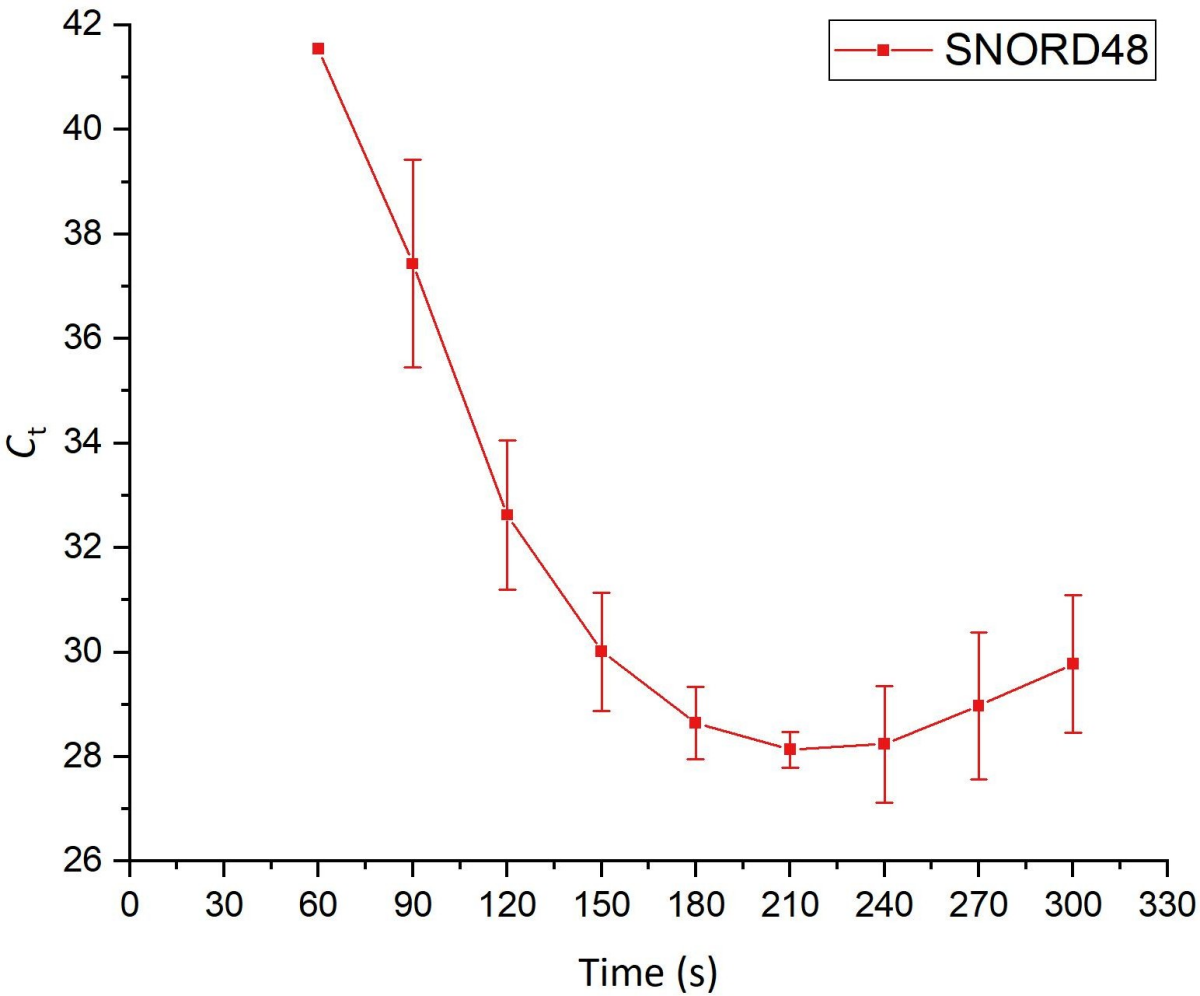
Time series showing the elution profile of the endogenous control RNA SNORD48 (57 b) from 5000 MCF7 cells (n = 3).

As most miRNA studies require normalization in respect to a stable reference RNA[22,23], co-extraction of the small nucleolar RNA (snoRNA) SNORD48 was attempted. A time series was performed and eluted fractions were collected every 30 seconds up to a total duration of 300 seconds (see Fig 11). SNORD48 was then detected by stem-loop RT-qPCR in the eluted fractions. The eluted amount of SNORD48 increases until a maximum is reached after 210 seconds after which the eluted amount decreases again. Similar to the data presented in Figs 3 and 4, these results show that by choosing a longer electrophoresis duration, co-elution of the target miRNAs and a longer endogenous control RNA was possible.

## Discussion

In previous work by our group on-chip thermoelectric lysis of MCF7 cells as well as on-chip electrophoresis of synthetic miRNAs was demonstrated on a device based on the same microfluidic technology as the device presented in this work [19]. We expand on this work by coupling these two principles to extract amplifiable miRNAs and a snoRNA from MCF7 cell suspensions.

First, the performance of the on-chip gel-electrophoresis was evaluated using synthetic miRNAs. The chip system demonstrated higher miRNA recovery than a state-of-the-art kit based on silica columns. The same experiment was carried out using synthetic snoRNA with both the chip and the silica columns performing similarly.

Next, using a breast cancer model (MCF7), the ability of the chip system to recover amplifiable miRNAs from cultured cells was investigated. Cells were introduced into the chip and lysed within 30 seconds by the application of a high-frequency voltage. Released miRNAs were then extracted from the crude lysate by on-chip gel-electrophoresis and miR16 as well as let7a were detected by stem-loop RT-qPCR. Interestingly, it was observed that the two investigated miRNA species were eluted from the crude lysate at different rates. This is quite unexpected, as both miRNAs are of the same length and are expected to migrate at similar rates during gel-electrophoresis. To rule out that the differential elution is caused by an intrinsic property of the chip, a miRNA sample obtained by kit extraction was subjected to on-chip gel-electrophoresis and the miR16/let7a ratio was measured before and after electrophoresis. The ratio was found to be unchanged after electrophoresis, proving that the chip does not preferentially elute a certain miRNA species.

A possible explanation for the observed behavior may be found in the fact that the chip based extraction system works under non-denaturing conditions due to the buffer and non-denaturing conditions in the hydrogel. Most cellular miRNAs are bound to protein complexes such as the RNA-induced silencing complex (RISC) [24,25] that catalyze the degradation of mRNA targets. Protein complexes that are still bound to the released miRNAs may thus influence the migration behavior during on-chip gel-electrophoresis.

To further investigate this theory, GEE was carried out with lysates obtained by TEL, off-chip heat shock and a combination of heat-shock followed by proteinase-K digest. The miR16/let7a ratio was then determined in the resulting extracts (Fig 8).

The miRNA samples obtained by TEL show the highest miR16/let7a ratio, indicating that either the elution of one of the investigated miRNA species is retarded or that the miRNA is rendered inaccessible to the reverse transcription reaction. The miRNA ratio obtained for the sample that was lysed off-chip by heat shock is already lower than that for on-chip lysis, hinting at further degradation of miRNA associated protein complexes. The sample that was treated by proteinase K shows an even lower ratio, confirming that the observed elution effects are indeed caused by proteins. The samples obtained by the highly denaturing mirVana kit procedure displays the lowest ratio of the two investigated miRNAs as likely most of the miRNA associated protein complexes have been degraded during purification.

Next, the performance of the chip system was compared to a commercial miRNA extraction kit for cell counts ranging from 5 to 500 cells total. The chip system and kit showed linear extraction behavior across the complete range of investigated cell counts. The amount of recovered miRNAs by the chip system in comparison to the kit was again found to be dependent on the investigated miRNA species. However, the performance of the chip system was shown to be about 220-fold better for the extraction of miR16 and about 14-fold better for the extraction of let7a.

To further demonstrate the versatility of the on-chip miRNA extraction system, we showed the extraction of the longer endogenous control snoRNA SNORD48 by increasing the duration of the on-chip gel-electrophoresis. This result shows that not only biomarker miRNAs but also the necessary controls may be obtained in a single step using the novel chip based approach.

In light of the fact that most miRNA function is based on its complexation with mRNA modifying proteins, a miRNA extraction system that is able to distinguish between complexed and uncomplexed miRNAs may be of great scientific interest. The presented microfluidic chip system could thus be employed to study the complexation of miRNAs during different cell states.

Further application of the chip system may include studies that investigate a wider panel of extracted miRNAs by either microarray or sequencing. The small size and ease of use may also open the chip approach for automation and parallelization.

The system may also be expanded to handle clinical samples or archive material such as formalin-fixed paraffin-embedded (FFPE) tissue.

By optimization of the chip design towards lower sample and elution volumes, we strongly believe that miRNA extraction from single cells will be possible.

## Methods

### Cell culture and miRNA extraction

MCF7 cells were cultivated from cryogenic stocks (BIOSS Toolbox, University of Freiburg, Germany) in DMEM supplemented with 10% fetal calf serum and 1% Penicillin-Streptomycin at 37°C, 5% CO_2_ and 95% humidity. After aspiration of the growth medium, cells were harvested by trypsinization and resuspension in phosphate buffered saline (PBS). Serial dilutions were prepared in PBS at a total volume of 2.4 ml using Neubauer chambers for cell counting. Final cell concentrations were 1x10^6^, 1x10^5^, 1x10^4^, 1000, 100 and 10 cells/ml. Finally, samples were split with one half being processed using the novel chip-based method and the second half being used for the kit-based extraction.

Kit extractions were performed using the mirVana (Ambion) miRNA extraction kit. Starting volumes were 1.2 ml PBS cell suspension. miRNAs were eluted into 100 μl DEPC treated water and stored at -20°C.

### RNA

Table 1 and Table 2 list the miRNA and snoRNA species used in this study. For brevity, abbreviations are used throughout the manuscript. Synthetic analogs of the RNAs were ordered from Bioron (Germany) and are denoted with “-(s)“.

**Table 1:** List of microRNA species used in this study.

miRBase ID	Abbreviation	miRBase Accession	Length (nt)
hsa-miR-16-5p	miR16	MIMAT0000069	22
hsa-let-7a-5p	let7a	MIMAT0000062	22

**Table 2:** Small nucleolar RNA used in this study.

Name	Accession	Length (nt)
SNORD48	NR_002745	57

### Device design and manufacture

The microfluidic devices are manufactured using a dry-film based photolithographic clean-room process described previously by Vulto et al.[18]. Briefly, platinum electrodes are structured on a Pyrex substrate using a lift-off process. Chamber walls and phaseguides are then formed by first laminating layers of Ordyl dry-film photoresist onto the substrates that are subsequently structured by photolithography.

A second Pyrex substrate with drilled holes is then thermally bonded onto the structured dry-film resist. Finally, individual chips are released by dicing.

The design of the microfluidic chip is identical to that presented in previous work by our group[26]. Briefly, the overall geometry and chamber volumes of the device, that was originally designed for RNA extraction from bacteria, were found suitable for the extraction of small RNAs from cultured cells. For a more detailed description of the design, the reader is referred to Hügle et al. [26].

To interface the chips with laboratory instrumentation, a custom chip holder is manufactured by CNC milling that provides electric as well as fluidic connectors.

### Device operation

First, a non-denaturing 5% polyacrylamide gel (PAG) solution is prepared by mixing 125 μl 40% 29:1 Acrylamide/Bisacrylamide solution, 867.5 μl 1X TBE, 2 μl TEMED and 5.5 μl 10% Ammonium persulfate. The solution is pipetted into the gel filling ports of the chips. The chips are then placed inside a small polymerization chamber that is constantly flushed with moisturized nitrogen. After gel polymerization is complete, the chips are either used immediately or sealed using PCR-plate adhesive foil and stored at 4°C for later use.

For each experiment, a chip is mounted into the custom holder. Using a standard laboratory pipette, the elution chamber (ec) is then filled with 1X TBE buffer and the sample chamber (sc) is filled with the sample without the addition of RNAse inhibitors. The electrode chambers are flushed with 1X TBE buffer at a flow rate of 200 μl/min by means of a syringe pump.

To perform lysis, a 20 kHz sine wave at a peak-to-peak voltage of 230 V_pp_ is applied to the electrodes. After lysis, the electrodes are connected to a DC voltage of 12 V for electrophoresis. Finally, the eluted miRNA samples are recovered from the elution chamber by pipetting and are immediately stored at -20°C.

Off-chip lysis was performed by incubating the cell suspension at 95°C for 30s. Proteinase K digestion was performed for one hour at 50°C at a proteinase K concentration of 200 μg/ml.

### Stem-loop RT-qPCR and data processing

First-strand cDNA synthesis of mature miRNAs was performed using the TaqMan microRNA reverse transcription kit (Applied Biosystems). A master mix containing reverse transcription (RT) buffer, miRNA specific stem-loop RT primer, dNTPs, RNAse inhibitor and Multiscribe reverse transcriptase was prepared according to the manufacturer’s instructions. 5 μl RT master mix were combined with 2.5 μl sample and incubated for 30 min. at 16°C, 30 min. at 42°C and 5 min. at 85°C for deactivation of the reverse transcriptase. For negative control reactions, the 2.5 μl sample were replaced by the same volume of PCR-grade water.

Quantitative PCR (qPCR) of the cDNA samples was carried out using TaqMan Universal PCR Master Mix (Applied Biosystems) on both LightCycler 1.5 (Roche) and LightCycler 480 (Roche) real-time PCR systems. Reactions were prepared at a final volume of 20 μl consisting of 10 μl 2X Universal PCR Master Mix, 1 μl primer/probe mix, 7.67 μl PCR-grade water and 1.33 μl cDNA. Thermal cycling was carried out inside plastic capillaries (GeneON) or 96 well plates beginning with a denaturation step of 95°C for 10 min. followed by 50 cycles of 95°C for 10 sec. and 60°C for 60 sec.

Fluorescence was read after each combined annealing/elongation step. Threshold cycle (*C*_t_) values were determined using the LightCycler data analysis software suite (Roche). Results with threshold cycles that were equal or greater than those of the negative control reactions were excluded from further analysis.

To enable valid comparison of the chip and kit experiments, raw threshold cycles (*C*_*tr*_) were processed to compensate for the different sample (*v*_*s*_) and elution (*v*_*e*_) volumes by the following equation:

$$C_{t}=C_{tr}+{log}_{2}(\frac{v_{s}}{v_{e}})$$ [1]

Table 3 gives an overview of the sample and elution volumes used for the chip and kit extractions.

**Table 3:** Sample and elution volumes used for the chip and kit extractions.

Method	*v*_*s*_ (μl)	*v*_*e*_ (μl)
Chip	5	10
Kit (Synthetic RNA)	100	100
Kit (Cell suspensions)	1200	100

All error-bars used throughout this work denote standard deviation.

### Recovery efficiency

To evaluate the performance of the on-chip gel-electrophoresis (GEE), synthetic miRNA (miR16-(s), 6.02x10^8^ copies/μl) and snoRNA (SNORD48, 6.02x10^8^ copies/μl) was first introduced into the sample chamber. A time-series was then performed by applying 12 V_DC_ to the electrodes for fixed time steps. After each time step, the contents of the elution chamber were recovered and the elution chamber was refilled with fresh 1X TBE buffer. The same synthetic miRNA was also subjected to the commercial mirVana miRNA extraction procedure.

The recovered miRNA fractions were then analyzed by stem-loop RT-qPCR and the fraction *f* of the initial concentration that was recovered was calculated by

$$f=\frac{6.02\cdot 10^{8}\mu l^{-1}}{2^{\left((C_{te}+{log}_{2}(\frac{v_{i}}{v_{e}}))-C_{ti}\right)}}$$ [2]

where *C*_*ti*_ and *C*_*te*_ are the threshold cycles measured before and after GEE and *v*_*i*_ and *v*_*e*_ are the initial and eluted sample volumes.

### Model fitting

Processed threshold cycle data was fitted to a first order polynomial of the form

$$C_{t}(n_{c})=m\cdot n_{c}+C_{t0}$$ [3]

with cell count *n*_*c*_ and fitting parameters *m* and *C*_*t*0_. The fold difference of the measured miRNA amount was subsequently calculated by taking the difference of the linear models:

$$Fold\;difference=2^{\left(C_{t1}(n_{c})-C_{t2}(n_{c})\right)}$$ [4]

## Supporting information

S1 Animated GIF Migration of fluorescently labeled DNA probe in electric field. (GIF)

S1 File Description of supplementary material. (DOCX)

## References

[1] Fromm B, Billipp T, Peck LE, Johansen M, Tarver JE, King BL, et al A Uniform System for the Annotation of Vertebrate microRNA Genes and the Evolution of the Human microRNAome. Annual Review of Genetics. 2015;49: 213–242. 10.1146/annurev-genet-120213-092023 26473382 PMC4743252

[2] Aravin A, Tuschl T. Identification and characterization of small RNAs involved in RNA silencing. FEBS letters. 2005;579: 5830–40. 10.1016/j.febslet.2005.08.009 16153643

[3] Calin G a, Croce CM. MicroRNA signatures in human cancers. Nature reviews Cancer. 2006;6: 857–66. 10.1038/nrc1997 17060945

[4] Xiong G, Feng M, Yang G, Zheng S, Song X, Cao Z, et al The underlying mechanisms of non-coding RNAs in the chemoresistance of pancreatic cancer. Cancer Letters. 2017;397: 94–102. 10.1016/j.canlet.2017.02.020 28254409

[5] Catto JWF, Alcaraz A, Bjartell AS, De Vere White R, Evans CP, Fussel S, et al MicroRNA in prostate, bladder, and kidney cancer: A systematic review. European Urology. 2011;59: 671–681. 10.1016/j.eururo.2011.01.044 21296484

[6] Zaporozhchenko IA, Morozkin ES, Skvortsova TE, Bryzgunova OE, Bondar AA, Loseva EM, et al A phenol-free method for isolation of microRNA from biological fluids. Analytical Biochemistry. 2015;479: 43–47. 10.1016/j.ab.2015.03.028 25843265

[7] Rajput SK, Dave VP, Rajput A, Pandey HP, Datta TK, Singh RK. A column-based rapid method for the simultaneous isolation of DNA, RNA, miRNA and proteins. Cell biology international. 2012;36: 779–83. 10.1042/CBI20110342 22553923

[8] Motameny S, Wolters S, Nürnberg P, Schumacher B. Next generation sequencing of miRNAs—Strategies, resources and methods. Genes. 2010;1: 70–84. 10.3390/genes1010070 24710011 PMC3960865

[9] Chen C, Ridzon D a, Broomer AJ, Zhou Z, Lee DH, Nguyen JT, et al Real-time quantification of microRNAs by stem-loop RT-PCR. Nucleic acids research. 2005;33: e179 10.1093/nar/gni178 16314309 PMC1292995

[10] Zhou D, Du W, Xi Q, Ge J, Jiang J. Isothermal Nucleic Acid Amplification Strategy by Cyclic Enzymatic Repairing for Highly Sensitive MicroRNA Detection. Analytical chemistry. 2014;86: 6763–7. 10.1021/ac501857m 24949808

[11] Arzt L, Kothmaier H, Quehenberger F, Halbwedl I, Wagner K, Maierhofer T, et al Evaluation of formalin-free tissue fixation for RNA and microRNA studies. Experimental and Molecular Pathology. 2011;91: 490–495. 10.1016/j.yexmp.2011.05.007 21641900

[12] Kramer MF. Stem-Loop RT-qPCR for miRNAs. Current Protocols in Molecular Biology. 2011;95: 1–15. 10.1002/0471142727.mb1510s95 21732315 PMC3152947

[13] Burgos KL, Javaherian A, Bomprezzi R, Ghaffari L, Rhodes S, Courtright A, et al Identification of extracellular miRNA in human cerebrospinal fluid by next-generation sequencing. RNA (New York, NY). 2013;19: 712–722. 10.1261/rna.036863.112 23525801 PMC3677285

[14] Redshaw N, Wilkes T, Whale A, Cowen S, Huggett J, Foy CA. A comparison of miRNA isolation and RT-qPCR technologies and their effects on quantification accuracy and repeatability. BioTechniques. 2013;54: 155–164. 10.2144/000114002 23477383

[15] Bravo V, Rosero S, Ricordi C, Pastori RL. Instability of miRNA and cDNAs derivatives in RNA preparations. Biochemical and biophysical research communications. 2007;353: 1052–5. 10.1016/j.bbrc.2006.12.135 17204243

[16] Schoch RB, Ronaghi M, Santiago JG. Rapid and selective extraction, isolation, preconcentration, and quantitation of small RNAs from cell lysate using on-chip isotachophoresis. Lab on a Chip. 2009;9: 2145 10.1039/b903542g 19606290

[17] Shintaku H, Nishikii H, Marshall L a, Kotera H, Santiago JG. On-Chip Separation and Analysis of RNA and DNA from Single Cells. Analytical Chemistry. 2014;86: 1953–1957. 10.1021/ac4040218 24499009

[18] Vulto P, Dame G, Maier U, Makohliso S, Podszun S, Zahn P, et al A microfluidic approach for high efficiency extraction of low molecular weight RNA. Lab on a chip. 2010;10: 610–616. 10.1039/b913481f 20162236

[19] Dame G, Lampe J, Hakenberg S, Urban G. Development of a Fast miRNA Extraction System for Tumor Analysis Based on a Simple Lab on Chip Approach. Procedia Engineering. 2015;120: 158–162. 10.1016/j.proeng.2015.08.593

[20] Vulto P, Podszun S, Meyer P, Hermann C, Manz A, Urban G a. Phaseguides: a paradigm shift in microfluidic priming and emptying. Lab on a Chip. 2011;11: 1596 10.1039/c0lc00643b 21394334

[21] Heneghan HM, Miller N, Kelly R, Newell J, Kerin MJ. Systemic miRNA-195 Differentiates Breast Cancer from Other Malignancies and Is a Potential Biomarker for Detecting Noninvasive and Early Stage Disease. The Oncologist. 2010;15: 673–682. 10.1634/theoncologist.2010-0103 20576643 PMC3228012

[22] Wong L, Lee K, Russell I, Chen C. Endogenous controls for real-time quantitation of miRNA using TaqMan microRNA assays. Applied Biosystems Application Note. 2010; 1–8. Available: http://scholar.google.com/scholar?hl=en&btnG=Search&q=intitle:Endogenous+Controls+for+Real-Time+Quantitation+?+of+miRNA+Using+TaqMan+MicroRNA+Assays.#1

[23] Gee HE, Buffa FM, Camps C, Ramachandran A, Leek R, Taylor M, et al The small-nucleolar RNAs commonly used for microRNA normalisation correlate with tumour pathology and prognosis. British Journal of Cancer. 2011;104: 1168–1177. 10.1038/sj.bjc.6606076 21407217 PMC3068486

[24] Gregory RI, Chendrimada TP, Cooch N, Shiekhattar R. Human RISC couples microRNA biogenesis and posttranscriptional gene silencing. Cell. 2005;123: 631–640. 10.1016/j.cell.2005.10.022 16271387

[25] Meister G, Landthaler M, Patkaniowska A, Dorsett Y, Teng G, Tuschl T. Human Argonaute2 mediates RNA cleavage targeted by miRNAs and siRNAs. Molecular Cell. 2004;15: 185–197. 10.1016/j.molcel.2004.07.007 15260970

[26] Hügle M, Dame G, Behrmann O, Rietzel R, Karthe D, Hufert FT, et al A lab-on-a-chip for preconcentration of bacteria and nucleic acid extraction. RSC Advances. 2018;8: 20124–20130. 10.1039/C8RA02177E PMC9080779 35541671

