# Supplementary figures and images for: A lab-on-a-chip for rapid miRNA extraction
Source: PLoS One. 2019 Dec 19;14(12):e0226571. doi: 10.1371/journal.pone.0226571 (PMC6922460; doi:10.1371/journal.pone.0226571)

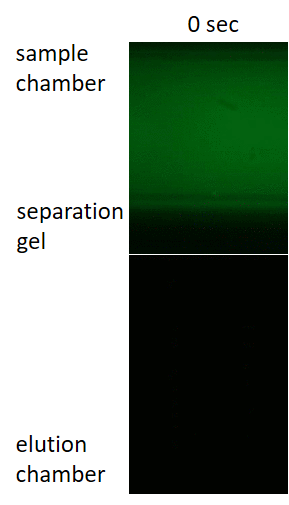

Supplement: S1 Animated GIF — (GIF) [file pone.0226571.s001.gif]
