# Supplementary material for: A lab-on-a-chip for rapid miRNA extraction
Source: PLoS One. 2019 Dec 19;14(12):e0226571. doi: 10.1371/journal.pone.0226571 (PMC6922460; doi:10.1371/journal.pone.0226571)
Supplement: S1 File — (DOCX) [file pone.0226571.s002.docx]

**Description of supplementary material**

We introduced a fluorescently labeled DNA probe into the sample chamber of the chip at a final concentration of 20µM. The chip was then placed on a fluorescence microscope and on-chip gel-electrophoresis was performed for 70 seconds at a voltage of 36 V_DC_ while pictures were taken at the time points indicated in the supplementary data file.
